# Eye Movements in Strategic Choice

**DOI:** 10.1002/bdm.1901

**Published:** 2015-10-29

**Authors:** Neil Stewart, Simon Gächter, Takao Noguchi, Timothy L. Mullett

**Affiliations:** ^1^ University of WarwickCoventryUK; ^2^ University of NottinghamNottinghamUK; ^3^ University College LondonLondonUK

**Keywords:** eye tracking, process tracing, experimental games, normal‐form games, prisoner's dilemma, stag hunt, hawk–dove, level‐*k*, cognitive hierarchy, drift diffusion, accumulator models, gaze cascade effect, gaze bias effect

## Abstract

In risky and other multiattribute choices, the process of choosing is well described by random walk or drift diffusion models in which evidence is accumulated over time to threshold. In strategic choices, level‐*k* and cognitive hierarchy models have been offered as accounts of the choice process, in which people simulate the choice processes of their opponents or partners. We recorded the eye movements in 2 × 2 symmetric games including dominance‐solvable games like prisoner's dilemma and asymmetric coordination games like stag hunt and hawk–dove. The evidence was most consistent with the accumulation of payoff differences over time: we found longer duration choices with more fixations when payoffs differences were more finely balanced, an emerging bias to gaze more at the payoffs for the action ultimately chosen, and that a simple count of transitions between payoffs—whether or not the comparison is strategically informative—was strongly associated with the final choice. The accumulator models do account for these strategic choice process measures, but the level‐*k* and cognitive hierarchy models do not. © 2015 The Authors. *Journal of Behavioral Decision Making* published by John Wiley & Sons Ltd.

When we make decisions, the outcomes that we receive often depend not only on our own choices but also on the choices of others. The related cognitive hierarchy and level‐*k* theories are perhaps the best developed accounts of reasoning in strategic decisions. In these models, people choose by best responding to their simulation of the reasoning of others. In parallel, in the literature on risky and multiattribute choices, drift diffusion models have been developed. In these models, evidence accumulates until it hits a threshold and a choice is made. In this paper, we consider this family of models as an alternative to the level‐*k*‐type models, using eye movement data recorded during strategic choices to help discriminate between these accounts. We find that while the level‐*k* and cognitive hierarchy models can account for the choice data well, they fail to accommodate many of the choice time and eye movement process measures. In contrast, the drift diffusion models account for the choice data, and many of their signature effects appear in the choice time and eye movement data.

## Level‐*k* Theory

Level‐*k* theory is an account of why people should, and do, respond differently in different strategic settings. In the simplest level‐*k* model, each player best responds assuming that everyone else is one level of reasoning behind them (Costa‐Gomes & Crawford, 2006; Nagel, 1995). To reason up to level *k* − *1* for other players means, by definition, that one is a level‐*k* player. A simple starting point is that level‐0 players choose randomly from the available strategies. A level‐1 player is assumed to best respond under the assumption that everyone else is a level‐0 player. A level‐2 player is assumed to best respond under the assumption that everyone else is a level‐1 player. More generally, a level‐*k* player best responds to a level *k* − *1* player. This approach has been generalized by assuming that each player chooses assuming that their opponents are distributed over the set of simpler strategies (Camerer et al., 2004; Stahl & Wilson, 1994, 1995). Thus, a level‐2 player is assumed to best respond to a mixture of level‐0 and level‐1 players. More generally, a level‐*k* player best responds based on their beliefs about the distribution of other players over levels 0 to *k* − *1*. By fitting the choices from experimental games, estimates of the proportion of people reasoning at each level have been constructed. Typically, there are few *k* = 0 players, mostly *k* = 1 players, some *k* = 2 players, and not many players following other strategies (Camerer et al., 2004; Costa‐Gomes & Crawford, 2006; Nagel, 1995; Stahl & Wilson, 1994, 1995).

These models make predictions about the cognitive processing involved in strategic decision making, and experimental economists and psychologists have begun to test these predictions using process‐tracing methods like eye tracking or Mouselab (where participants must hover the mouse over information to reveal it). What sort of eye movements or lookups are predicted by a level‐*k* strategy?

### Information acquisition predictions for level‐*k* theory

**Figure 1:**
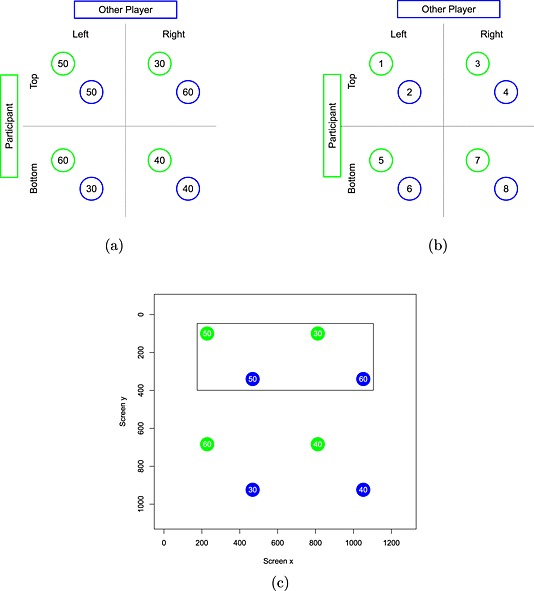
(a) An example 2 × 2 symmetric game. This game happens to be a prisoner's dilemma game, with top and left offering a cooperating strategy and bottom and right offering a defect strategy. The row player's payoffs appear in green. The column player's payoffs appear in blue. (b) The labeling of payoffs. The player's payoffs are odd numbers; their partner's payoffs are even numbers. (c) A screenshot from the experiment showing a prisoner's dilemma game. In this version, the player's payoffs are in green, and the other player's payoffs are in blue. The player is playing rows. The black rectangle appeared after the player's choice. The plot is to scale, with axes indicating screen coordinates in pixels

We illustrate the predictions of level‐*k* theory with a 2 × 2 symmetric game taken from our experiment (Figure 1a). Two players must each choose a strategy, with their payoffs determined by their joint choices. We will describe games from the point of view of a player choosing between top and bottom rows who faces another player choosing between left and right columns. For example, in this game, if the row player chooses top and the column player chooses right, then the row player receives a payoff of 30, and the column player receives 60.

**Figure 2:**
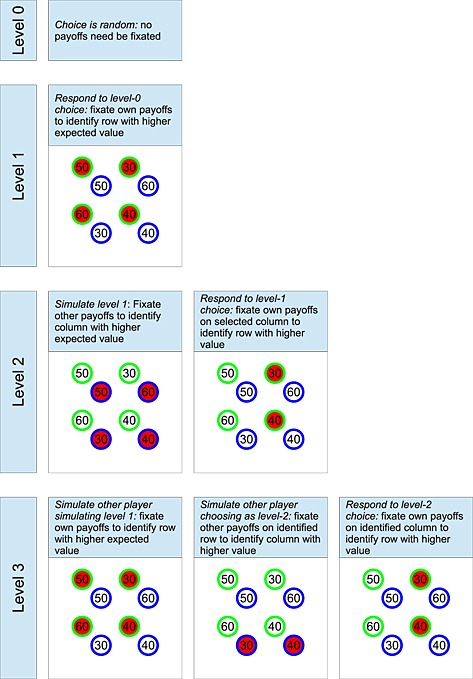
Eye movements expected in level‐*k* theory, illustrated for levels 0–3. At each stage, relevant payoffs are highlighted in red. The illustration is for a particular prisoner's dilemma game, the fourth in Table 2

Figure 2 illustrates the payoff information needed at each stage for different levels of level‐*k* reasoning, following Costa‐Gomes, Crawford, and Broseta (2001). A level‐0 player chooses randomly and could do this with his or her eyes closed! A level‐1 player best responds to the random choice of a level‐0 player. This means that he or she must view his or her own payoffs, highlighted in red in the level‐1 row of Figure 2, to select the action with the highest expected payoff. A level‐2 row player must first simulate the column player using level‐1 reasoning. A level‐1 column player will look up his or her own payoffs and determine which column offers the higher expected payoff under the assumption of a level‐0 row player choosing a row randomly. Having identified the choice of his or her level‐1 column playing opponent, the player must then look up his or her own payoffs for that column to select a row. Thus, a level‐2 player should first examine the other player's payoffs and then examine one column of his or her own payoffs. A level‐3 player first examines his or her own payoffs as they simulate the other player at level‐2 simulating them as a level‐1 player. Then they examine the other player's payoffs for the action the other player thinks they themselves will take. Finally, having identified how the other player will choose, they examine their own payoffs for that action.

## Assumptions Relating Theory and Process Measurements

In the previous section, a number of assumptions relating the level‐*k* theory to information acquisition patterns were implicit. Table 1 lists the most common assumptions made by researchers, which we review later in detail. In doing so, we summarize previous research on process tracing in strategic choice, which has focussed upon looking for process patterns that discriminate between level‐*k*, learning, and rational choice models.

**Table 1:** Assumptions about eye movements in strategic choice made by previous researchers

Assumption	Source
Information acquisition	
For a model to fit, all necessary payoffs must be viewed.	Costa‐Gomes et al. (2001), Devetag et al. (2015), and Knoepfle et al. (2009)
Looking at unnecessary information counts as evidence against a model.	Costa‐Gomes et al. (2001) and Knoepfle et al. (2009)
Attention	
The number/durations of fixations of a payoff indicate attention to that payoff.	Costa‐Gomes et al. (2001), Johnson et al. (2002), Knoepfle et al. (2009), and Wang et al. (2010)
People re‐fixate rather than remember payoffs.	Costa‐Gomes et al. (2001)
Transitions	
Order—fixations to payoffs involved in later stages only count as hits if they occur after all of the fixations required for earlier stages.	Camerer et al. (1993), Chen et al. (2011), Johnson et al. (2002), and Knoepfle et al. (2009)
Adjacency—comparisons of payoffs appear as a fixation to the first payoff immediately followed by a fixation to the second payoff.	Costa‐Gomes et al. (2001) and Devetag et al. (2015)

The least controversial assumption is that people must view the payoffs, which are used in the model. People cannot be making a decision using information that they have not viewed. For example, Costa‐Gomes et al. (2001) score models as complying when all payoffs used in the model are viewed but do not penalize a model if it fails to predict observed viewings of payoffs which are not used by the model.

Some researchers make the further assumption that viewing information that is not required by a model is evidence against that model. For example, Knoepfle, Wang, and Camerer (2009) explored learning in normal‐form games by comparing adaptive learning models against strategic choice models. The eye movement data show that players looked at their opponent's payoffs about as often as their own payoffs. The opponent's payoffs play no role in the adaptive learning models, and Knoepfle et al. conclude that viewing the opponent's payoffs is evidence against the adaptive models and evidence in favor of the strategic models. Knoepfle et al. also construct a hit rate measure—the proportion of fixations to payoffs required by a model. Fixations to non‐required payoffs reduce the hit rate and count against the model.

One step beyond simply measuring whether required payoffs are viewed is to take the number of lookups or their duration as a measure of attention to that payoff. This is common in eye movement studies of other types of decision (in risky choice, e.g., Stewart, Hermens, & Matthews, 2015, or in consumer choice, e.g., Krajbich, Armel, & Rangel 2010). In strategic choice, Wang, Spezio, and Camerer (2010) tracked the eye movements of senders in a sender–receiver game, finding that senders attended to the true action too much and failed to take the perspective of the receiver, who was ignorant of the true action. In another example, Costa‐Gomes et al. (2001) use the number of lookups of the different types of payoff (e.g., the player's versus his or her opponents) as diagnostic of type of the player (Table 4).

Memory is a costly activity—remembering even a small set of numbers is hard (e.g., Miller, 1956). So while a player could simply read each payoff in the game once and then make the decision based entirely on that memory, this is probably not what is happening. In strategic choice, payoffs are often revisited multiple times (Costa‐Gomes et al., 2001), just as they are in risky choice even for simple gambles (Stewart, Hermens, & Matthews, 2015) and in choices between familiar snacks (Krajbich et al., 2010). It is cognitively cheaper to make a reacquisition eye movement than try to remember.

As people refixate rather than remember, the sequencing of lookups of payoffs can be used to discriminate between models. Knoepfle et al. (2009) recorded eye movements in 4 × 4 normal‐form games, imposing “a simple order restriction requiring at least one lookup in a stage's lookup area before lookups in the next stage's area count as hits” (p. 396). Johnson, Camerer, Sen, and Rymon (2002) also used weak constraints in the ordering of lookups to test whether people used backwards induction in a three‐round sequential bargaining game. Chen, Huang, and Wang (2011) used the ordering of transitions to identify a player's *k* level in a spatial beauty contest.

While the previous examples involve weak assumptions about the sequence of lookups, inferences are often made from pairs of temporally adjacent lookups where one immediately follows the other. For example, in risky choice, consecutive lookups of probability and then amount within a gamble are taken as evidence for an expected value calculation, whereas consecutive lookups of the amount in one gamble and then the amount in another gamble, for example, are taken as evidence of a trading off between amounts (Russo & Dosher, 1983; see Stewart, Hermens, & Matthews, 2015, for a review). Similar assumptions are made in multiattribute choice (Noguchi & Stewart, 2014). Indeed, instructing people to trade off or calculate expectations changes the proportions of these consecutive lookups (Arieli, Ben‐Ami, & Rubinstein, 2011), which is strong causal evidence that different consecutive lookups result from different strategies.

Costa‐Gomes et al. (2001) made use of consecutive lookups to identify the *k* level of their participants. Their adjacency criteria required that “each comparison in some minimal set needed to identify a [level‐*k*] type's decision is represented by an adjacent look‐up pair at least once in the subject's look‐up sequence” (p. 1210). That is, if a model requires a comparison between a pair of payoffs, those payoffs should appear next to one another at some point in the ordered sequence of payoffs viewed. Devetag, Di Guida, and Polonio (2015) also assumed that consecutive lookups indicate comparisons of those payoffs in their 3 × 3 games.

## Current Conclusions from Process Data in Games

Having constructed Table 1, we can summarize the key conclusions that have emerged from those who have used normal‐form games. Costa‐Gomes et al. (2001) conclude that Mouselab lookups and choices were most consistent with level‐1 and level‐2 models, with no participants classified as best responding either as rational maximizers. Knoepfle et al. (2009) explored learning in normal‐form games and found that although the adaptive learning models fitted choice behavior best but not eye movements, whereas level‐*k*‐like models fitted eye movements best but not choices. Devetag et al. (2015) used 3 × 3 normal‐form games and conclude that players are behaving as if they make level‐1 choices or select obvious focal points.

Beyond these normal‐form game studies, other types of strategic scenarios have been considered. As described previously, Camerer, Johnson, Rymon, and Sen (1993) and Johnson et al. (2002) used lookups revealed by Mouselab to conclude that untrained players do not use backwards induction in a three‐round sequential bargaining game. Wang et al. (2010) concluded from eye movements in sender–receiver games that senders choose as if they have different levels of *k* making eye movements consistent with those levels (and see Chen et al., 2011, for a similar agreement in a spatial beauty contest). To sum up thus far, perhaps one conclusion can be drawn: under minimal assumptions, eye movements are more consistent with level‐*k* reasoning with *k* = 1 or 2 than they are with the rational model.

A second approach taken by experimenters is to compare eye movements in strategic decisions with the eye movements of control groups instructed to follow certain strategies. This approach neatly side steps the issue of making assumptions about which eye movements are to be expected for certain cognitive processes. For example, in addition to the analysis described previously, Costa‐Gomes et al. (2001) taught some players game theory including how to use dominance, iterated dominance, dominance solvability, and pure strategy equilibrium. These trained participants made different eye movements, making more comparisons of payoffs across a change in action than the untrained participants. These differences suggest that, without training, participants were not using methods from game theory (see also Funaki, Jiang, & Potters, 2011).

## Accumulator Models

**Figure 3:**
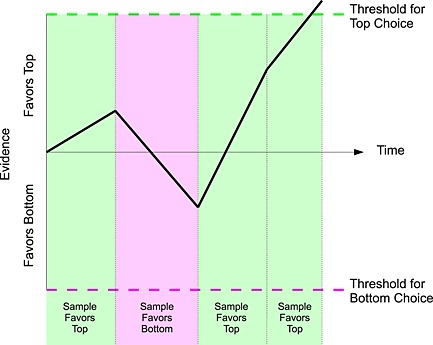
An example accumulator model

Accumulator models have been extremely successful in the domains of risky choice and choice between multiattribute alternatives like consumer goods. Figure 3 illustrates a basic but quite general model. The bold black line illustrates how the evidence for choosing top over bottom could unfold over time as four discrete samples of evidence are considered. The first, third, and fourth samples provide evidence for choosing top, while the second sample provides evidence for choosing bottom. The process finishes at the fourth sample with a top response because the net evidence hits the high threshold. We consider exactly what the evidence in each sample is based upon in the following discussions. In the case of the discrete sampling in Figure 3, the model is a random walk, and in the continuous case, the model is a diffusion model.

Perhaps people's strategic choices are not so different from their risky and multiattribute choices and could be well described by an accumulator model. In risky choice, Stewart, Hermens, and Matthews (2015) examined the eye movements that people make during choices between gambles. Among the models that they compared were two accumulator models: decision field theory (Busemeyer & Townsend, 1993; Diederich, 1997; Roe, Busemeyer, & Townsend, 2001) and decision by sampling (Noguchi & Stewart, 2014; Stewart, 2009; Stewart, Chater, & Brown, 2006; Stewart, Reimers, & Harris, 2015; Stewart & Simpson, 2008). These models were broadly compatible with the choices, choice times, and eye movements. In multiattribute choice, Noguchi and Stewart (2014) examined the eye movements that people make during choices between non‐risky goods, finding evidence for a series of micro‐comparisons of pairs of alternatives on single dimensions as the basis for choice. Krajbich et al. (2010) and Krajbich and Rangel (2011) have developed a drift diffusion model that, by assuming that people accumulate evidence more rapidly for an alternative when they fixate it, is able to explain aggregate patterns in choice, choice time, and fixations. Here, rather than focus on the differences between these models, we use the class of accumulator models as an alternative to the level‐*k* accounts of cognitive processes in strategic choice.

While the accumulator models do not specify exactly what evidence is accumulated—although we will see that the difference in payoffs across actions is a good candidate—the models do make some key predictions about eye movements. Assuming that the evidence for an alternative is accumulated faster when the payoffs of that alternative are fixated, accumulator models predict more fixations to the alternative ultimately chosen (Krajbich et al., 2010). Because evidence is sampled at random, accumulator models predict a static pattern of eye movements across different games and across time within a game (Stewart, Hermens, & Matthews, 2015). But because evidence must be accumulated for longer to hit a threshold when the evidence is more finely balanced (i.e., if steps are smaller, or if steps go in opposite directions, more steps are required), more finely balanced payoffs should give more (of the same) fixations and longer choice times (e.g., Busemeyer & Townsend, 1993). Because a run of evidence is needed for the difference to hit a threshold, a gaze bias effect is predicted in which, when retrospectively conditioned on the alternative chosen, gaze is made more and more often to the attributes of the chosen alternative (e.g., Krajbich et al., 2010; Mullett & Stewart, 2015; Shimojo, Simion, Shimojo, & Scheier, 2003). Finally, if the nature of the accumulation is as simple as Stewart, Hermens, and Matthews (2015) found for risky choice, the association between the number of fixations to the attributes of an action and the choice should be independent of the values of the attributes.

To preempt our results, the signature effects of accumulator models described previously appear in our eye movement data. That is, a simple accumulation of payoff differences to threshold accounts for both the choice data and the choice time and eye movement process data, whereas the level‐*k* and cognitive hierarchy models account only for the choice data.

## The Present Experiment

In the present experiment, we explored the choices and eye movements made by participants in a range of symmetric 2 × 2 games. Our approach is to build statistical models, which describe the eye movements and their relation to choices. The models are deliberately descriptive to avoid missing systematic patterns in the data that are not predicted by the contending theories, and so our more exhaustive approach differs from the approaches described previously (see also Devetag et al., 2015). We are extending previous work by considering the process data more deeply, beyond the simple occurrence or adjacency of lookups.

## Method

### Participants

Fifty‐four undergraduate and postgraduate students were recruited from Warwick University and participated for a payment of £5 plus a further payment of up to £5 contingent upon the outcome of a randomly selected game. For four additional participants, we were not able to achieve satisfactory calibration of the eye tracker. These four participants did not begin the games. Participants provided written consent in line with the institutional ethical approval.

## Apparatus

Stimuli were presented on an LCD monitor viewed from approximately 60 cm with a 60‐Hz refresh rate and a resolution of 1280 × 1024. Eye movements were recorded with an Eyelink 1000 desk‐mounted eye tracker (SR Research, Mississauga, Ontario, Canada), which has a reported average accuracy between 0.25° and 0.50° of visual angle and root mean square resolution of 0.01° (www.sr‐research.com). We tracked participants' right eye movements using the combined pupil and corneal reflection setting at a sampling rate of 500 Hz. Head movements were tracked, although we used a chin rest to minimize head movements.

### Games

Each participant completed the sixty‐four 2 × 2 symmetric games, listed in Table 2. The *y* columns indicate the payoffs in £. Payoffs are labeled 1–8, as in Figure 1b. The participant's payoffs are labeled with odd numbers, and the other player's payoffs are labeled with even numbers. Games were symmetric, so the column player's payoffs are a transpose of the row player's payoffs (i.e., *y*_1_ = *y*_2_, *y*_3_ = *y*_6_, *y*_5_ = *y*_4_, and *y*_7_ = *y*_8_).

**Table 2:**
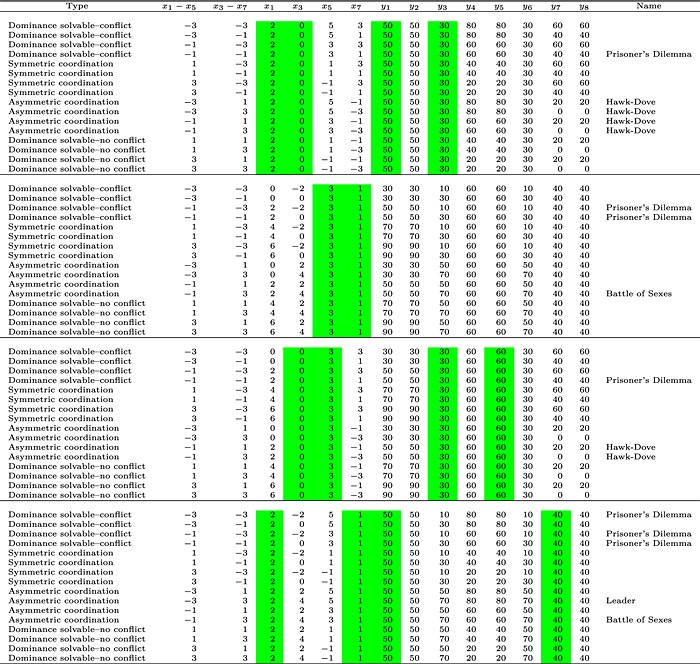
Games used

*Note*: Actual payoffs in £ are given by *y*_1_ − *y*_8_. The *x* columns define the games (as described in the main text), with the *y* payoffs given by multiplying by £10 and adding £30. Highlighting indicates the payoffs that were held constant, with other payoffs generated using *x*_1_ − *x*_5_ and *x*_3_ − *x*_7_.

The *x* columns indicate how we generated the 64 games, as follows. *x*_1_, *x*_3_, *x*_5_, and *x*_7_ define the player's payoffs, with the actual *y* payoffs generated from the *x* values by multiplying by £10 and adding £30 so that payoffs were in the range £0–£90. For 2 × 2 symmetric games, games can be mapped onto a set of strategically equivalent games in two‐dimensional space (Weibull, 1995). The dimensions are defined by *x*_1_ − *x*_5_ and *x*_3_ − *x*_7_ (given in the second and third columns of Table 2). *x*_1_ − *x*_5_ is the difference in payoffs available to the player when their opponent chooses left. *x*_3_ − *x*_7_ is the difference in payoffs available to the player when their opponent chooses right.

So that we can explore how eye movements vary across games, we varied *x*_1_ − *x*_5_ and *x*_3_ − *x*_7_ systematically, with each difference taking values from {−3, − 1, 1, 3} creating 16 (*x*_1_ − *x*_5_, *x*_3_ − *x*_7_) pairs in a 4 × 4 grid. With *x*_1_ − *x*_5_ and *x*_3_ − *x*_7_ set, we need to fix one of *x*_1_ and *x*_5_ and one of *x*_3_ and *x*_7_ to define a game. For top 16 games in Table 2, we fixed *x*_1_ and *x*_3_ or, equivalently, *y*_1_ and *y*_3_. The green highlight indicates the fixed payoffs. Subsequent sets of 16 games were generated using the same (*x*_1_ − *x*_5_, *x*_3_ − *x*_7_) pairs but with other *x*s fixed.

Defining games in the (*x*_1_ − *x*_5_, *x*_3_ − *x*_7_) space is useful because the type of the game depends on the signs of these two differences. Dominance solvable–conflict games, when *x*_1_ − *x*_5_ < 0 and *x*_3_ − *x*_7_ < 0, are dominance solvable and have a conflict between cooperation and maximizing one's own payoff and include some prisoner's dilemma games. Symmetric coordination games, when *x*_1_ − *x*_5_ > 0 and *x*_3_ − *x*_7_ < 0, include some stag hunt (or assurance) games. Asymmetric coordination games, when *x*_1_ − *x*_5_ < 0 and *x*_3_ − *x*_7_ > 0, include hawk–dove (or chicken or snowdrift), battle‐of‐the‐sexes, and leader games. Dominance solvable–no conflict games, when *x*_1_ − *x*_5_ > 0 and *x*_3_ − *x*_7_ > 0, are dominance solvable but with no conflict between cooperation and maximizing one's own payoff.

### Stimuli

Figure 1c shows how games were presented. To avoid complicating eye movements, the display was as simple as possible. The presentation of payoffs in a small font and circle ensures that participants cannot read one payoff while fixating another and must make an eye movement. In this screenshot, the player is playing rows, with their payoffs highlighted in green and the other player's payoffs highlighted in blue. The black rectangle appeared post‐response and indicated whether the player chose, in this case, top or bottom. Between participants, we counterbalanced whether the participant played rows or columns, whether the participant's payoffs were green or blue, and whether the participant's payoffs appeared in the top left or bottom right of each cell. Randomly, for each presentation of each game, we swapped rows top to bottom and columns left to right.

### Procedure

Participants were seated in front of the experiment computer and eye tracker. Participants were shown an example game. Written instructions explained how one player was selecting rows and the other columns and how each player would receive the payoff at the intersection of the chosen row and column. Horizontal and vertical black rectangles appeared (like the one in Figure 1c) to indicate the intersection. Participants then received a practice trial and were encouraged to ask the experimenter, who was present throughout the experiment, any questions. Participants were told that, after all participants had been tested, participants would be paired up, a random game selected, and outcomes paid according to their choice and the other player in their pair. Payments were subject to an experiment exchange rate, and participants could win up to £5.

Each trial began with a drift correction fixation cross, before a game appeared. Row players pressed the up or down cursor key to indicate their choice. Column players used the left and right keys. No information about the other player's choices was given. A 13‐point calibration was used initially and every 10 trials to maintain accuracy. Participants were encouraged to stretch and be comfortable before each calibration. The experiment typically took about 30 min to complete.

## Results

We have recoded results so that we can describe the data in terms of a participant who was making row choices, had their payoffs in green, and had their payoffs in the top left of each cell and received games with rows and columns ordered as in Figure 1b.

In the eye movement data, each fixation was classified as being to a particular payoff if it fell within a 100‐pixel‐radius circle of the center of the payoff. This crude classification produces almost identical results to a maximum likelihood assignment of fixations to clusters for each payoff (Stewart, Hermens, & Matthews, 2015).

### Choices

**Figure 4:**
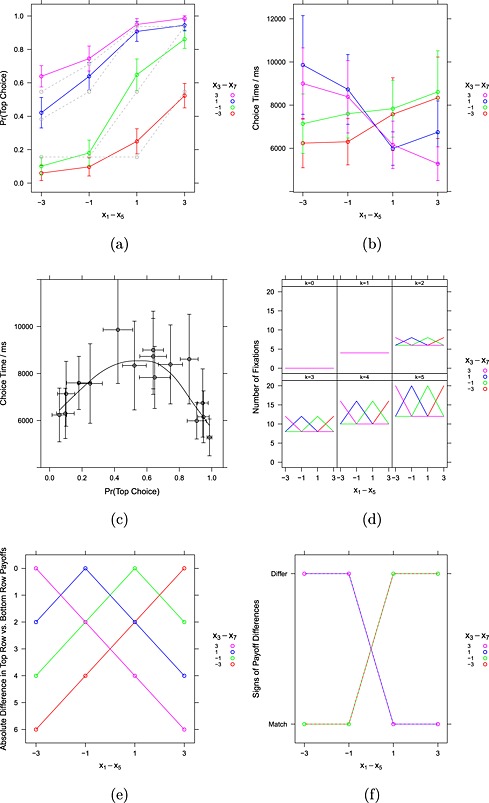
(a) The proportion of “top” choices as a function of *x*_1_ − *x*_5_ and *x*_3_ − *x*_7_. The gray lines show the best‐fitting predictions from a level‐*k* model with a mixture of levels 0, 1, and 2 participants. (b) Choice time as a function of *x*_1_ − *x*_5_ and *x*_3_ − *x*_7_. (c) Choice time as a function of the proportion of “top” choices. (d) Predictions of a level‐*k* model for the number of fixations required for a decision. (e) Absolute difference in top‐row and bottom‐row payoffs. (f) Do *x*_1_ − *x*_5_ and *x*_3_ − *x*_7_ match in sign? Error bars are 95% confidence intervals

Figure 4a shows how choices varied across games by plotting the proportion of top choices as a function of the differences *x*_1_ − *x*_5_ and *x*_3_ − *x*_7_. Larger differences make the payoffs on the top row larger and make players more likely to choose top. A logistic mixed effects regression that predicts choice as a function of *x*_1_ − *x*_5_, *x*_3_ − *x*_7_, and their interaction, necessarily including full random slopes, estimates no meaningful interaction, odds ratio = 0.99, 95% confidence interval (CI) [0.96, 1.02], but large and about equal effects of *x*_1_ − *x*_5_, odds ratio = 2.3, 95% CI [2.1, 2.5], and *x*_3_ − *x*_7_, odds ratio = 2.5, 95% CI [2.2, 2.8]. Thus, the differences *x*_1_ − *x*_5_ and *x*_3_ − *x*_7_, which capture the strategic differences between games, capture the differences in player's choices across games well.

The variation in choice proportions is large. In the dominance solvable–conflict games (*x*_1_ − *x*_5_ < 0 and *x*_3_ − *x*_7_ < 0), which includes some prisoner's dilemma games, people almost always select bottom. This level of defection is high, but compared with the other games, cooperation in these games is relatively unappealing (Vlaev & Chater, 2006). In dominance solvable–no conflict games (*x*_1_ − *x*_5_ > 0 and *x*_3_ − *x*_7_ > 0) where top is the dominant strategy, offering the highest outcomes irrespective of the other player's choice, people almost always choose top. Choice proportions are intermediate for the other games. Table 3 tracks the key results, of which this is the first.

**Table 3:** A summary of key results

Result	Level‐*k*	Accumulator
Higher top‐row payoffs increase top‐row choices.	✓Good fit	✓Good fit
Choices take longer, the closer choice proportions are to .5.	✕Only predicts that games requiring a mixed strategy (where (*x*_1_ − *x*_5_) = − (*x*_3_ − *x*_7_)) take longer	✓Predicts that games where the signs of *x*_1_ − *x*_5_ and *x*_3_ − *x*_7_ agree should be faster
Players fixate their own payoffs more than the other player's.	✓But only odd *k* predicts an own‐payoff bias	–No prediction
Within‐cell, within‐row, and within‐column transitions are all frequent, with a higher frequency of within‐row transitions between the player's payoffs.	✕Does not predict any within‐cell transitions. Does not predict frequent within‐row transitions between the player's payoffs	✓Higher‐frequency within‐row own‐payoff transitions follow assuming integration of payoffs within a row to form the drift rate
Larger payoffs are fixated a little more often.	✕Only predicts more fixations when (*x*_1_ − *x*_5_) = − (*x*_3_ − *x*_7_)	–No prediction
A bias to fixate the payoffs on the ultimately chosen row develops over the course of a choice.	✕No gaze bias	✓The gaze bias is a signature effect in accumulator models
Transitions to a row predict choice of that row …	✕ Predicts that transitions are independent of choice	✓Assuming evidence for an option is accumulated at a higher rate when that option is fixated
… whether or not they are informative.	✕Predicts that dumb transitions are not informative	✓Assuming evidence for an option is accumulated at a higher rate when that option is fixated

#### Level‐*k* choices

For each game, the level‐*k* model predicts a choice of the top row, the bottom row, or a random selection. Table 4 lists the predictions for each *x*_1_ − *x*_5_ and *x*_3_ − *x*_7_, as predictions are the same for games with matching *x*_1_ − *x*_5_ and *x*_3_ − *x*_7_. For these games, odd‐numbered levels (1, 3, 5, 7, …) predict the same choices, and even numbered levels except 0 (2, 4, 6, 8, …) predict the same choices.

**Table 4:** Level‐*k* choice predictions

*x*_1_ − *x*_5_	*x*_3_ − *x*_7_	Level‐*k* prediction		
		Level 0	Level 1	Level 2
−3	−3	Guess	Bottom	Bottom
−3	−1	Guess	Bottom	Bottom
−3	1	Guess	Bottom	Top
−3	3	Guess	Guess	Guess
−1	−3	Guess	Bottom	Bottom
−1	−1	Guess	Bottom	Bottom
−1	1	Guess	Guess	Guess
−1	3	Guess	Top	Bottom
1	−3	Guess	Bottom	Bottom
1	−1	Guess	Guess	Guess
1	1	Guess	Top	Top
1	3	Guess	Top	Top
3	−3	Guess	Guess	Guess
3	−1	Guess	Top	Top
3	1	Guess	Top	Top
3	3	Guess	Top	Top

The level‐*k* fit to the choice proportions is shown as a dashed line in Figure 4a. To fit the level‐*k* model, we have estimated the predictions of a mixture of different *k* levels. The best fitting mixture proportions were 19.5% level 0, 54.8% level 1, and 25.7% level 2. The level‐*k* model captures the qualitative pattern in the choice data quite well. These proportions match those reviewed in the Introduction, with mostly level‐1, few level‐0, and few level‐2.

#### Accumulator choices

Accumulator models fit the choice proportion effects well too. For example, in the drift diffusion model, the probability of a choice is a logit function of the drift rate (e.g., Bogacz, Brown, Moehlis, Holmes, & Cohen, 2006). Here, we consider the difference in payoffs for the top and bottom rows, (*x*_1_ − *x*_5_) + (*x*_3_ − *x*_7_), as the drift rate. But this is just the form for the logistic regression used previously to model how choice proportions varied over games, and so with a straightforward assumption about the evidence accumulated, the accumulator models account for choice data quite naturally.

### Choice times

Figure 4b plots choice time (from game onset to keypress) as a function of *x*_1_ − *x*_5_ and *x*_3_ − *x*_7_. Where the differences *x*_1_ − *x*_5_ and *x*_3_ − *x*_7_ have the same sign (i.e., point towards the same row), people are faster. Figure 4c, where choice time is plotted against the choice proportions from Figure 4a, makes the pattern in choice times obvious: choice times are longest for games where choice proportions are most finely balanced, and choice times are shortest for games where there is a strong preference either for top or for bottom. Because, as we see next, the duration of individual fixations is pretty much constant across games and time course, this means that choice time is extremely strongly correlated with the number of fixations per choice (*r*=.98, 95% CI [.95, .99]) and thus that the number of fixations shows the same relationship with choice proportions.

#### Choice times

The most straightforward way to consider choice time predictions for level‐*k* is to assume that choice time is proportional to the number of payoffs required for a decision. For example, Figure 2 shows that, for a level‐2 decision, six payoffs must be looked up and compared.

Figure 4d plots the number of fixations predicted by level‐*k*. Higher levels require more lookups, and, when *k* = 0 or 1, all games require the same number of fixations, and, for *k* ≥ 2, more fixations are required when (*x*_1_ − *x*_5_) = − (*x*_3_ − *x*_7_) because, in these cases, simulated strategies for the lower levels involve the need to consider the best response to a mixed strategy. No matter what proportions that we assume for the different levels of *k*, the level‐*k* model fails to capture the pattern in the choice time data, as noted in Table 3.

#### Accumulator choice times

In accumulator models, choices take longer when the evidence for each alternative is more finely balanced. In the previous discussions, we considered a drift rate of (*x*_1_ − *x*_5_) + (*x*_3_ − *x*_7_) to explain the choice data. Rearranged, the drift rate is (*x*_1_ + *x*_3_) − (*x*_5_ + *x*_7_), which is the top payoffs less the bottom payoffs. The absolute value of this difference is plotted in Figure 4e. Zero differences, when evidence is most finely balanced, are plotted at the top, because these should correspond to the slowest times. Larger differences, when the evidence clearly points in one direction, are plotted at the bottom, because these should be faster. But a modification of this prediction is informative. Figure 4f notes whether the differences *x*_1_ − *x*_5_ and *x*_3_ − *x*_7_ have the same sign or not. When they agree in sign, both are evidence in the same direction—either both point to a top choice or both point to a bottom choice. When they differ in sign, one comparison favors a top‐row choice, and the other favors a bottom‐row choice. People should be faster when signs match, and so matching has been plotted at the bottom of the plot. By collapsing together *x*_1_ − *x*_5_ and *x*_3_ − *x*_7_ differences of the same sign—for this is the difference between Figure 4e and 4f—the qualitative pattern is choice time predictions quite close to the data in Figure 4b.

### Fixation durations

The average duration of a fixation was 290 milliseconds. Such brief fixations are typically associated with automatic rather than deliberative processing (Fiedler & Glöckner, 2012; Horstmann, Ahlgrimm, & Glöckner, 2009, but see Su et al., 2013).

We examined how fixation durations varied over games. A mixed effects model of fixation duration as a function of the *x*_1_ − *x*_5_ difference, the *x*_3_ − *x*_7_ difference, and their interaction, which necessarily included full random effects, shows that fixation durations hardly vary at all across games with unit changes in *x*_1_ − *x*_5_, *x*_3_ − *x*_7_, and their interaction all affecting durations by at most only 2 milliseconds ( $\beta_{x_{1}\;-\;x_{5}}=-0.2$ milliseconds, 95% CI [−0.8, 0.3]; $\beta_{x_{3}\;-\;x_{7}}=-0.5$ milliseconds, 95% CI [−1.1, 0.1]; and $\beta_{\left(x_{1}\;-\;x_{5}\right)\;\times \;\left(x_{3}\;-\;x_{7}\right)}=0.24$ milliseconds, 95% CI [0.0, 0.5]).

Fixation durations are also constant over the time course of a trial. A mixed effects model of fixation duration as a function of fixation number, which necessarily included full random effects for fixation duration, shows that each successive fixation is only 2.6 milliseconds faster, 95% CI [1.7, 3.3].

Fixation durations are important in the analysis of reading, because variation in their duration indicates differences in processing (Rayner, Pollatsek, Ashby, & Clifton, 2012). The stability of duration here across games and over the time course of a choice suggests constant cognitive processes across and throughout choices.

### Fixation and transition frequencies

**Figure 5:**
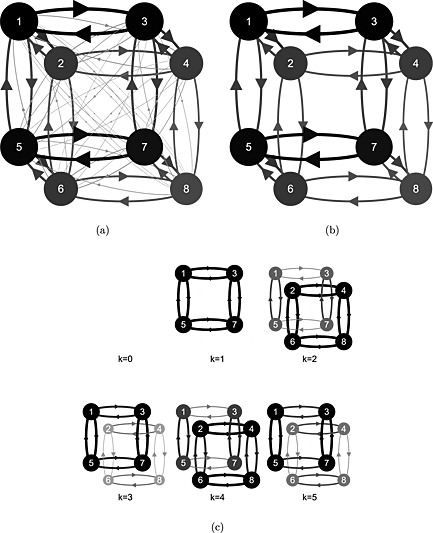
(a) Fixation and transition frequencies. (b) As (a) but with rare transitions omitted for clarity. (c) Level‐*k* predictions for fixation and transition frequencies. The area and blackness of the circles at the payoffs indicate the fixation frequencies. The thickness and blackness of arrows indicate the transition frequencies

Immediately in the following text, we describe the pattern of fixation and transition frequencies. Afterward, we present a statistical estimation confirming this pattern. The mean, across participants, of the number of fixations made per game is 17, which is enough to fixate each payoff about twice. Figure 5a displays the frequencies of fixations to each payoff and the frequencies of transitions between those payoffs. The area and blackness of the circles at each payoff are proportional to the frequency of fixation, and the larger darker circles for *y*_1_, *y*_3_, *y*_5_, and *y*_7_ compared with *y*_2_, *y*_4_, *y*_6_, and *y*_8_ indicate that players fixate their own payoffs a little more often than the other player's. We note this in Table 3.

#### Two types of transition: common and rare

The thickness and blackness of the arrows in Figure 5a are proportional to the frequency of transitions between payoffs. It is useful to consider two categories of transition we will call *common* and *rare*—theory agnostic labels based on frequency. Common transitions involve exactly one change in either the payoff's owner (e.g., *y*_1_ → *y*_2_), the player's action (e.g., *y*_1_ → *y*_5_), or other player's action (e.g., *y*_1_ → *y*_3_). Figure 5b repeats Figure 5a, displaying only the common transitions and omitting the rare transitions. Rare transitions involve multiple changes (e.g., *y*_1_ → *y*_8_, where the payoff owner and both actions change). Common transitions make up 76% of all transitions; rare transitions are the remaining 24%. On average, any given common transition is 5.3 times more frequent than any given rare transition.

Common transitions could be useful comparisons. For example, transitions within a cell where only the owner of the payoff changes (e.g., *y*_1_ → *y*_2_) could be useful if people have other‐regarding preferences. Transitions where only the player's action changes (e.g., *y*_1_ → *y*_5_) could be useful for calculating the difference in payoffs for each row. Transitions where only the other player's action changes (e.g., *y*_1_ → *y*_3_) contain information about how the player's payoff changes if the other player switches action. It is harder to tell a story about the use to which rare transitions could be put. Too many things are changing at once. But some proportion of rare transitions is to be expected as people switch between comparisons.

Considering the common transitions, the darker, thicker arrows between *y*_1_ and *y*_3_ and between *y*_5_ and *y*_7_ in Figure 5b show that players make frequent eye movements between their payoffs within a row. That is, players compare the payoffs they will receive across the two actions of the other player. Other common transition frequencies are smaller and about equal. We note this pattern in Table 3.

#### A Poisson regression for fixation and transition frequencies

To describe the fixation and transition frequencies, we have fitted them using a mixed effects Poisson regression with full random effects. We used 24 dummy variables to code the properties of each of the transitions in Figure 5b. The model is saturated—there are 24 coefficients that fit the 24 transition frequencies without error. As fixation frequencies are an aggregation over transition frequencies, these are also modeled. Thus, the Poisson regression provides an exhaustive analysis of the fixation frequencies and their first‐order sequential dependence. This approach contrasts with considering only the subset of patterns predicted by existing theories and ensures that we do not miss any systematic pattern. Stewart, Hermens, and Matthews (2015) provide a complete description of this approach as applied to risky choice.

The coefficients are displayed in the first column of Table 5 (ignore later columns for now). We have presented exponentially transformed coefficients because, in Poisson regression, frequencies are given by the products of transformed coefficients. The *intercept* coefficient of 20.64 represents the overall number of transitions made by a player. (Summed over all 64 games, 20.64 is the geometric mean, across participants, of the geometric mean number of times that the transitions illustrated in Figure 5b were made.) This means that each of these transitions is made about once every three games.

**Table 5:**
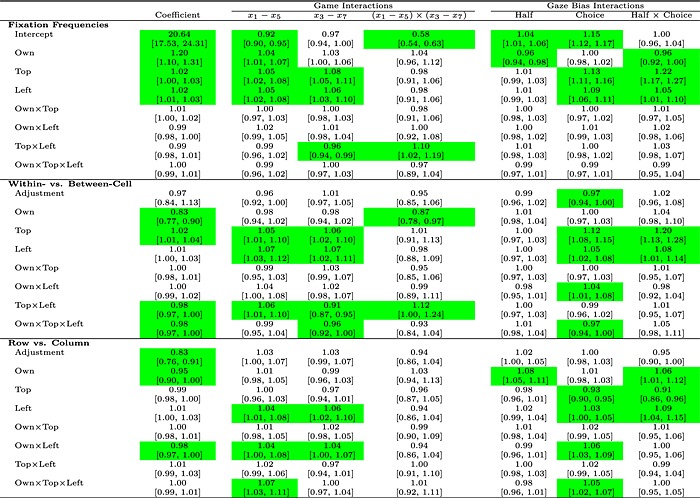
Exponentially transformed coefficients and their 95% confidence intervals for the saturated model of the transition matrix

*Note*: Coefficients have been exponentially transformed. Coefficients with confidence intervals that do not contain 1 are highlighted.

The next seven coefficients in the “Fixation Frequencies” section of Table 5 describe how the fixation frequencies differ over payoffs. Using the variable *own*, we dummy coded each region as +1 if it was the player's own payoff and −1 if it was the other player's payoff. The coefficient for *Own* at 1.20 means that the base frequency must be increased by a factor of 1.2 to obtain the frequency for fixations to the player's payoffs and divided by 1.2 to the frequency for fixations to the other player's payoffs. Thus, the proportion of fixations to the player's own payoffs is 1.2/(1.2 + 1 / 1.2) = 0.59. The coefficients for the six remaining dummies are all small, as demonstrated by the limits of the 95% CIs, making no more than a 3% change to fixation counts (i.e., coefficients are between 0.97 and 1.03): players have no tendency to fixate any row or column more often than another (the *left* and *top* coefficients), and there is no interaction between the owner and location of the payoff.

The remaining 16 coefficients in the first column, in the “Within vs. Between‐Cell” and “Row vs. Column” sections, describe how the foregoing fixation frequencies vary as a function of the location of the previous fixation. The eight “Within vs. Between‐Cell” coefficients are adjustments to the fixation frequencies depending on whether the transition is within a cell between payoffs (e.g., *y*_1_ ↔ *y*_2_) or a between‐cells change in (only one) action (e.g., *y*_1_ ↔ *y*_3_ or *y*_1_ ↔ *y*_5_). For example, the *own* coefficient in this section at 0.83 indicates that bias described previously for a player to fixate his or her own payoffs is reduced by a factor of 0.83 when the transition is between the payoffs within a cell (e.g., *y*_1_ ↔ *y*_2_) and increased by a factor of 1/0.83 when the transition is not within a cell (e.g., *y*_1_ ↔ *y*_3_ or *y*_1_ ↔ *y*_5_). The eight “Row vs. Column” coefficients are adjustments to transition frequencies depending on whether the transition is within columns (e.g., *y*_1_ ↔ *y*_5_) or within rows (e.g., *y*_1_ ↔ *y*_3_). For example, the *adjustment* coefficient at 0.83 indicates that within‐column transitions are less frequent than within‐row transitions. The 95% CIs for the others among these 16 coefficients indicate that the other effects are all small. This confirms the pattern in Figure 5b described previously: transitions across columns are more frequent than transitions within cells or transitions across rows. Devetag et al. (2015) also find this result.

Table 5 omits the coefficients for modeling the transitions in our rare category, which involve more than one change (e.g., *y*_1_ ↔ *y*_4_ or *y*_1_ ↔ *y*_8_). We did not find any notable patterns in the coefficients we have omitted.

#### Level‐*k* fixations and transitions

Figure 5c gives the fixation and transition frequencies predicted by level‐*k*. For the fixation frequency predictions, we assumed that each payoff required at each stage is looked up once. For the transition frequency predictions, we assumed that all transitions across rows or columns (but not both) within a stage, in any order, are possible. For example, for the *k* = 2 case in Figure 2, we assume that all transitions between the other player's payoffs are possible in the stage “Simulate level 1” (i.e., *y*_2_ ↔ *y*_4_, *y*_2_ ↔ *y*_6_, *y*_4_ ↔ *y*_8_, and *y*_6_ ↔ *y*_8_), and then all transitions between the level‐1 choice column are possible in the stage “Respond to level‐1 choice” (i.e., *y*_3_ ↔ *y*_7_). Thus, we do not make assumptions about adjacency as strong as those of Devetag et al. (2015) and Costa‐Gomes et al. (2001), because we are allowing any ordering of fixations between payoffs within a stage of level‐*k* reasoning. Source code is available.

Figure 5c shows that level‐*k* does predict an own‐payoff bias when *k* is odd. When *k* is even, the bias reverses. But, because in fitting choice data, the proportion of level‐1 required was higher than the proportion of level‐2, level‐*k* predicts a net bias to fixate own payoffs more. So although the model could also predict the reverse bias, we have logged this as a success for level‐*k* in Table 3.

Figure 5c also shows that the level‐*k* model, or any blend of level‐*k*s, misses the pattern of transitions. First, the model never predicts within‐cell transitions (there are no diagonal arrows). The within‐cell transitions suggest incorporating with other‐regarding preferences in the level‐*k* model. Second, the model does not predict the higher frequency of between‐column transitions between the player's own payoffs.

#### Accumulator models and fixations and transitions

In fitting choice and choice time data, we assumed a drift rate based on the difference in the payoffs in each row of (*x*_1_ − *x*_5_) + (*x*_3_ − *x*_7_). So we would expect the player's payoffs to be fixated equally often. It is less clear how a bias to fixate the player's own payoffs more than the other player's follows. Because the games are symmetric, the information in *x*_1_, *x*_3_, *x*_5_, and *x*_7_ is repeated in the other player's payoffs, and so any bias is consistent with our earlier assumptions. We log no clear prediction in Table 3.

### Do fixation counts and transition probabilities change as payoffs change?

The “Game Interactions” columns in Table 5 show how the fixation and transition frequencies change across games as *x*_1_ − *x*_5_ and *x*_3_ − *x*_7_ vary. We constructed a second mixed effects Poisson regression including (*x*_1_ − *x*_5_), (*x*_3_ − *x*_7_), (*x*_1_ − *x*_5_) × (*x*_3_ − *x*_7_), and their interactions with the original 24 dummy variables. The payoff differences were scaled so that coefficients represent the effect of payoff differences changing from minimum to maximum. The top *Intercept* row with coefficients 0.92, 0.97, and 0.58 shows how the number of fixations varied across games. Some games have nearly twice as many fixations as others. Because fixation counts are so highly correlated with choice time, as we described earlier, we have already seen this effect as games with more finely balanced choice proportions taking longer and thus more fixations (recall Figure 4b). In particular, the 0.58 coefficient indicates that when *x*_1_ − *x*_5_ and *x*_3_ − *x*_7_ are either both large (+3) or both small (−3), choices are fast because both differences point in the same direction.

The remaining coefficients in the “Fixation Frequencies” rows of the “Game Interactions” columns indicate how the distribution of fixations varies across games. The coefficients are all small, and the limits of the 95% CIs mean that we can say that variations in *x*_1_ − *x*_5_ and *x*_3_ − *x*_7_ made no more than a 10% difference in fixation counts. The *Own*, *Top*, and *Left* rows show that players looked a little more at larger payoffs. There is also a tendency for players to look more at the leading diagonal when *x*_1_ − *x*_5_ and *x*_3_ − *x*_7_ were either both large or both small. But to a first approximation, players fixated payoffs equally often across games.

The coefficients in the “Within‐ vs. Between Cell” and “Row vs. Column” rows of Table 5 describe how transition frequencies vary across games. All of these interactions are small, typically making no more than a 10% difference in transition counts. For example, we have already seen that players are less likely to make transitions to their own payoffs if the transition is within a cell rather than across rows or columns (see the foregoing discussions; the 0.83 coefficient in the *Own* row of the “Within‐ vs. Between Cell” section). The 0.87 value for the interaction of the *Own* dummy and (*x*_1_ − *x*_5_) × (*x*_3_ − *x*_7_), which appears in the “Within‐ vs. Between Cell” rows of the (*x*_1_ − *x*_5_) × (*x*_3_ − *x*_7_) column, indicates that this effect is even stronger when *x*_1_ − *x*_5_ and *x*_3_ − *x*_7_ agree in sign. But the overall pattern is for only small variation in transition frequencies across games. To sum up how eye movements vary across games, players make nearly twice as many eye movements on some games compared with others, but the type of eye movements they make changes very little across games—players just do more of the same eye movements on more balanced games.

### Do fixation counts and transition probabilities change over the time course of a single trial?

**Figure 6:**
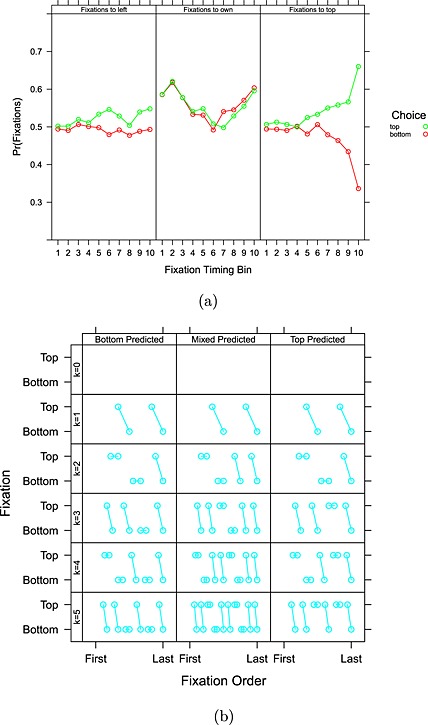
(a) The development of a bias towards fixating *own*, *left*, and *top* payoffs over time by choice. Fixations were binned into deciles, with early fixations in the first bin and the fixations at choice in the last. (b) Level‐*k* predictions for the gaze bias effect. Rows plot predictions for different levels of *k*. Columns break predictions down by the predicted choice

The final columns headed “Gaze Bias Interactions” of Table 5 evaluate how fixations and transitions depend on what people ultimately choose and how this effect emerges over the time course of a trial. We ran a third Poisson regression with dummy variables indicating whether a transition was in the first or second half of the fixation sequence, whether the choice was top row or bottom row, their interaction, and the interactions with the original 24 dummy variables. The *Intercept* row is uninteresting and just indicates that there are more fixations in the second half of a trial (because the middle fixation was arbitrarily assigned) and slightly more trials where the top action was selected. The only effect in the “Fixation Frequencies” section is that people make more fixations to the payoffs in the row that they ultimately choose and that this pattern develops over time. Figure 6a indicates this clearly. The last panel plots the proportion of fixations to the top payoffs as a function of time conditioned by the action chosen. On trials where top is ultimately chosen, a bias for top locations develops from about halfway through a trial. Similarly, a bias against top locations (i.e., for bottom locations) develops if bottom is ultimately chosen. This is the classic gaze bias effect (Fiedler & Glöckner, 2012; Shimojo et al., 2003; Stewart, Hermens, & Matthews, 2015), noted in Table 3. Other biases are much smaller.

The sections “Within‐ vs. Between Cell” and “Row vs. Column” show exactly which transitions change in frequency to create the overall gaze bias. Breaking the gaze cascade effect down into transitions, when top is ultimately chosen, the transitions that increase are the top‐row transitions, and the transitions that decrease are the bottom‐row transitions.

#### Level‐*k* and the gaze bias effect

Level‐*k* does not predict the gaze bias effect. Figure 6b plots the sequence of fixations predicted by level‐*k*, which were calculated by simulating out the process in Figure 2 and plotting out fixation location over time as a function of the chosen row. In level‐*k*, for all *k* > 0, the last pair of fixations is across rows. This means that level‐*k* is predicting no overall bias for the top or bottom row in the last two fixations, which is not consistent with the bias to fixate the ultimately chosen row being strongest at this point. We note this in Table 3.

#### Accumulator models and the gaze bias effect

Mullett and Stewart (2015) demonstrated that the gaze bias effect is a signature of accumulator models with a difference‐based stopping rule. For example, to reach the threshold for an option in a drift diffusion model, a run of evidence is needed for one option over the other. Under the assumption that evidence is accumulated at a higher rate for the fixated option (Krajbich et al., 2010), this means that when conditioned on choice, there should be a run of fixations to the attributes of the chosen option leading up to the choice. Thus, as we note in Table 3, the gaze bias effect is an inevitable prediction of the accumulator models, providing that a difference‐based stopping rule is used.

### Differences between row and column players

We have also explored the differences in the transitions made by row versus column players (which we counterbalanced between participants) by including a dummy variable for orientation and interactions with this dummy in the Poisson regression. The only notable difference is that, for between‐cell transitions, players like to make more horizontal than vertical eye movements, which means that row players make more transitions across the actions of the other player than column players do.

### Choice from eye movements

Because we expect eye movements to be related to cognitive processing, we expect there to be an association between the choice that people ultimately make and their eye movements (see also Devetag et al., 2015; Stewart, Hermens, & Matthews, 2015). Table 6 explores this, listing the accuracy with which choices can be modeled. Each model is a simple logistic regression, fitting the choice on a trial from various properties of the payoffs, fixations, or transitions. The intercept model describes the fact that 56% of choices were top.

**Table 6:** Accuracy with which choices can be fitted based on choice attributes, eye movements, or both

Model	Accuracy	BIC	Nagelkerke *R* ^2^
Intercept	.56	4658	.00
*choice* ∼1			
Attributes	.80	3085	.56
*choice* ∼1 + (*x*_1_ − *x*_5_) * (*x*_3_ − *x*_7_) * *base*			
Fixations	.67	4285	.15
*choice* ∼1 + *F*_1_ + *F*_2_ + · + *F*_8_			
Attributes and fixations	.83	2928	.60
*choice* ∼1 + (*x*_1_ − *x*_5_) * (*x*_3_ − *x*_7_) * *base* + *F*1 + *F*2 + · + *F*8			
Last fixation	.70	4094	.21
*choice* ∼1 + *Flast*			
Transitions	.71	4112	.27
*choice* ∼1 + *T*12 + *T*13 + · · + *T*_78_			
Transitions and attribute values	.85	2835	.66
*choice* ∼1 + (*x*_1_ − *x*_5_) * (*x*_3_ − *x*_7_) * *base* + *T*_12_ + *T*_13_ + · + *T*_78_			

*Note*: Schwartz's Bayesian information criterion (BIC) values are corrected for the nesting of choices within subjects. The BIC values show that better fitting models do provide a better account of the data and that the extra model parameters are warranted. In the *R*‐style regression equations, *choice* is a dummy variable for top versus bottom, 1 indicates that an intercept was included, payoff differences (*x*_1_ − *x*_5_) and (*x*_3_ − *x*_7_) were included as factors so that they were coded with a dummy for each payoff difference, *base* is a set of dummies indicating which quarter of Table 2 games came from,* indicates main effects of each term and interactions, *F_i_* is the frequency of fixations to payoff *i*, and *T_ij_* is the frequency of transitions from payoff *i* to *j*.

The attributes model uses *x*_1_ − *x*_5_, *x*_3_ − *x*_7_, a dummy for which of the *x*s was the base pair, and all of the interactions between these to fit choices. The model allows separate coefficients for each level of *x*_1_ − *x*_5_ and *x*_3_ − *x*_7_ (rather than a single slope), effectively allowing free functional forms for people's sensitivity to these payoff differences and their interactions. This model uses a separate coefficient for each of the 64 games. No other choice model could do better. This model achieves an accuracy of 80.0%. But this flexibility is not warranted—a model with only single slope coefficients for *x*_1_ − *x*_5_ and *x*_3_ − *x*_7_ and no interactions (i.e., *choice* ∼ 1 + (*x*_1_ − *x*_5_) + (*x*_3_ − *x*_7_), where (*x*_1_ − *x*_5_) and (*x*_3_ − *x*_7_) entered as numerical and not factors)—achieves accuracy of 79.6%, and is preferred by Schwartz's Bayesian information criterion (BIC). Note that BIC values were corrected for the nesting of choices within subjects.

We described earlier that the accumulator models predict choice probabilities as a logit function of drift rates. That the aforementioned simple logit model, with only *x*_1_ − *x*_5_ and *x*_3_ − *x*_7_ and no interactions, is preferred over the much more complicated model means that the choice data do not support assuming anything more complicated than the difference in payoffs between rows is driving the evidence accumulation.

**Figure 7:**
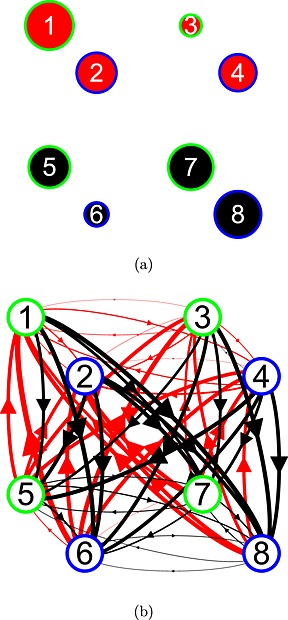
(a) Coefficients for fitting choices from the number of fixations to each region. Red indicates positive coefficients (top choices more likely), and black indicates negative coefficients (bottom choices more likely). The area of the circles indicates the magnitude of the coefficients. (b) Coefficients for fitting choices from the number of transitions between regions. The width of the arrows indicates the magnitude of the coefficients

The fixations model uses only the counts of fixations to each location to fit choice and achieves accuracy of 67.3%. Figure 7a plots the best‐fitting coefficients. Fixations to top‐row locations increase the likelihood of a top choice. Fixations to bottom‐row locations increase the likelihood of a bottom choice. Actually, an improper model, where all coefficients are constrained to take the same magnitude (i.e., *choice* ∼ 1 + *I*((*F*_1_ + *F*_2_ + *F*_3_ + *F*_4_) − (*F*_5_ + *F*_6_ + *F*_7_ + *F*_8_)), where the *I*() identity function aggregates the difference in fixations so that a single slope is used in this model), achieves 66.9% accuracy, and is preferred by BIC. In essence, all that matters is how often participants looked at the top versus the bottom locations.

The attributes and fixations model fits better than either the attribute model or the fixations models and is preferred by BIC demonstrating that combining eye movement data with payoff data improves the ability to fit choices.

The last fixation model uses only the location of the last fixation and fits choices with accuracy 70.0%. Recall from the discussion of the gaze bias effect that people have an emerging tendency to fixate the row that they ultimately choose. Note that the fixations model, which does not have any information about the ordering of fixations, is doing only slightly worse in accuracy.

The transitions model uses the transition frequencies for each trial to fit the choice on that trial. The model achieves 71.1% accuracy and, despite its extra complexity, is preferred by BIC over the fixations model (but not the really simple last fixation model, which does nearly as well in terms of accuracy). Figure 7b plots the coefficients for each transition. The size of a coefficient is the effect of one transition of that type on the likelihood of a top‐row choice, and transitions with a stronger effect are drawn with thicker arrows. Color indicates the direction of the effect. It is the between‐row transitions that matter (within‐row and within‐cell transitions have very small coefficients). Including only the between‐row transitions gives an accuracy of 64.9%. And improper modeling, where between‐row coefficients are constrained to have the same magnitude (i.e., $\begin{array}{l} \text{choice}∼1+I((T_{51}+T_{61}+T_{71}+T_{81}+T_{52}+T_{62}+T_{72}+T_{82}+T_{53}+T_{63}+T_{73}+T_{83}+T_{54}+T_{64}+T_{74}+\\ T_{84})-\left(\left(T_{15}+T_{25}+T_{35}+T_{45}+T_{16}+T_{26}+T_{36}+T_{46}+T_{17}+T_{27}+T_{37}+T_{47}+T_{18}+T_{28}+T_{38}+T_{48}\right)\right)))\text{,} \end{array}$fits nearly as well with an accuracy of 62.9% and is preferred by BIC because of its increased simplicity. Note that the transitions that are important for fitting are the between‐row transitions, but the transitions that increased over time to give the gaze bias effect described previously were the within‐row transitions for the selected row.

We have also taken into account the association between the difference in between‐row transition counts and the location of the last fixation. For example, if starting from the top row, the number of transitions to the top row from the bottom row minus the number of transitions to the bottom row from the top row must, necessarily, be 0 or −1. When the difference is 0, one is back where one started, and so the last fixation must be to the top row. When the difference is −1, the last fixation must be to the bottom row. By simply entering the last fixation into the regression before the transition frequencies, we have corrected the transition frequency coefficients for the last fixation. Although the coefficients are all a little smaller in magnitude, the pattern in Figure 7b remains.

Overall, transitions to a row increase the likelihood of a choice of that row (coefficients for transitions to *y*_1_ − *y*_4_ are positive; coefficients for transitions to *y*_5_ − *y*_8_ are negative). An interesting feature of the corrected coefficients (and the uncorrected coefficients) is that many rare‐category transition frequencies are associated with the choice even though the comparison they are associated with is uninformative. For example, *y*_1_ ↔ *y*_6_ transitions involve a swap between payoffs for the player and the other player and simultaneously a swap of action by the player. Even though this direct comparison is not informative for strategy selection, it is associated with choice. Of the 15 transitions, which have a strong effect on choice, 11 are rare‐category transitions.

It is useful to consider how much of the variance in choices is fitted by attribute values alone, transitions alone, and by both together. The Nagelkerke pseudo‐*R* ^2^ measure is used because it is additive, with a value of zero indicating that no variance is explained and a value of one indicating that choices are perfectly fitted. The Nagelkerke *R* ^2^ values are reported in Table 6. For the attribute model, Nagelkerke *R* ^2^=.56. For the transitions model, Nagelkerke *R* ^2^=.27. If attributes and transitions were each making a unique contribution, the combined Nagelkerke *R* ^2^ would therefore be .56+.27=.83, .17 higher than the actual Nagelkerke *R* ^2^=.66 for the transitions and attributes model. This means that about two‐thirds (.17/.27) of the variance explained by transitions is also explained by attribute values. Or, equivalently, about one‐third (.17/.56) of the variance explained by attribute values is also explained by transitions. Using the fixations models instead, we can say that about one‐sixth of the variation in choice explained by attributes is explained by fixations, and two‐thirds of the variation in choices explained by fixations is also explained by attribute values. So some, but by no means all, of the processing of payoffs is picked up by eye movements. And some of the effect of eye movements has an effect on choice independent of the attribute values.

## The Interaction between Eye Movements and Payoffs in Fitting Choices

The last thing that we explored was whether the effect on an eye movement on choice varied depending on the magnitude of the payoff fixated. In risky choice, Stewart, Hermens, and Matthews (2015) surprisingly found that this was not the case: looking at larger or more probable payoffs had the same effect as looking at smaller or less likely payoffs. Here, we find that fixation frequency × payoff value interactions are small. Including the interaction in the attributes and fixations model improves accuracy in fitting choices by 0.6%, although this small improvement is preferred by BIC. Because the cumulative effect of these interactions is only a very small improvement in accuracy, we do not discuss it further.

## General Discussion

Our participants played a set of 2 × 2 symmetric games where the payoffs were systematically varied to create dominance‐solvable games and symmetric‐ and asymmetric‐coordination games, including prisoner's dilemma, stag hunt, and hawk–dove games. We tracked participants' eye movements while they chose and explored whether and how eye movements varied within the time course of a choice and across the different types of games. In a second wave of modeling, we explored the relationship between eye movements and choice. We close with a summary of the core results and their implications for the level‐*k* and accumulator models.

Our players were very sensitive to the type of game presented, with choice proportions varying over a large range and systematically with the payoffs. Players clearly engaged with our games and differentiated between games in their choice behavior. A level‐*k* model that assumed a mixture of levels 0, 1, and 2 participants (or strategies within a participant) captured much of the variation in choice proportions. But the accumulator model, which predicted choice proportions as a logistic function of the difference in payoffs across rows, also fitted well.

Choice times were very strongly related to choice proportions, such that for games where the difference in payoffs across rows was more finely balanced, choice proportions were nearer 0.5, and choice times were much longer. This pattern is ubiquitous (e.g., Busemeyer & Townsend, 1993; Mosteller & Nogee, 1951; Petrusic & Jamieson, 1978). The level‐*k* model is unable to predict this choice time pattern, but it is a natural consequence of the accumulator framework in which finely balanced evidence means lower drift rates and thus longer times to threshold.

Individual fixation durations were brief, about 290 milliseconds, and unaffected by the game in question. Such brief fixations are typically associated with automatic processing as in accumulator models. But brief fixations are not consistent with a literal deliberative calculation of strategy as assumed by the level‐*k* and rational models.

The stability of fixation duration over the time course of a choice together with the stability of the pattern of eye movements over the time course does not offer any evidence of changes in cognitive processing over time. This suggests, for example, that there is not a reading phase (which would be associated with relatively brief fixations) followed by a deliberative calculation phase (which would be associated with slower fixations). Instead, this stability is more consistent with a constant processing over time, as in accumulator models.

Within a game, each payoff is fixated equally often, except for a small bias towards fixating one's own payoffs rather than the other players (also found by Devetag et al., 2015; Hristova & Grinberg, 2005; Knoepfle et al., 2009; Tanida & Yamagishi, 2010; Wang et al., 2010). Level‐*k* can predict this bias for odd levels of *k*. The accumulator account does not make a prediction as the framework does not make a clear prediction about how attention is distributed—although the pattern is not inconsistent with the model.

The pattern of eye movements varied only a little across games with a small bias to fixate the larger payoffs. Devetag et al. (2015) also only find small differences across games and Stewart, Hermens, & Matthews, 2015 see this in risky choice. The level‐*k* and accumulator models do not predict that larger payoffs will be fixated more.

Transitions between payoffs—that is breaking the fixations down contingent upon the immediately preceding fixation—reveal that people are making the eye movements that have been associated with the meaningful comparison of payoffs (Arieli, Ben‐Ami, & Rubinstein, 2009a, 2009b; Costa‐Gomes et al., 2001; Knoepfle et al., 2009). By far, the most frequent transitions are within a cell comparing the player's payoff with the other player's payoff, or are vertical eye movements comparing a pair of corresponding payoffs when the player swaps from top to bottom, or are horizontal eye movements comparing a pair of payoffs when the other player swaps between left and right. We called these the common transitions. Transitions where more than one of these things changes at once were rare. We argued that, because common transitions contain useful information, their high frequency seems like strong evidence that players understood the games and what sorts of comparisons might be useful in solving them.

Within the common transitions, transitions between the player's own payoffs under a change of action by their opponent were particularly frequent (see also Devetag et al., 2015). The level‐*k* model was unable to predict this and also fails to predict within‐cell transitions. The pattern is not inconsistent with the accumulator model driven by the net difference in payoffs between rows.

Over the time course of a choice, the pattern of eye movements is quite stable. Devetag et al. (2015) report stability in 3 × 3 games, and Funaki et al. (2011) report stability in three‐person dictator games. An exception to the stability of eye movements over time is the developing bias to fixate the payoffs of the row ultimately chosen, which emerged from about halfway through a choice. This gaze bias effect is ubiquitous and is seen in choice between consumer products, risky gambles, and even in choosing between attractive faces (Fiedler & Glöckner, 2012; Krajbich et al., 2010; Shimojo et al., 2003; Stewart, Hermens, & Matthews, 2015). The level‐*k* model predicts no gaze bias effect, because the last two fixations are always a between‐row comparison of a pair of payoffs as the participant finally selects a row choice given their inference about the other player's column choice. But the gaze bias effect is a signature of an accumulator model with a difference‐based stopping rule (Mullett & Stewart, 2015).

The small differences in eye movements across games are sufficient to fit choices reliably (see also Devetag et al., 2015). Using the size of the payoffs and no eye movement information allows choices to be fitted with about 80% accuracy. Using eye movement information and no payoff information allows choices to be fitted with about 70% accuracy. Together, eye movements and payoff information allow accuracy of about 85%. Fixations to the top row increase the probability of choosing the top row, and fixations to the bottom row decrease the probability of choosing the top row. Constraining all fixations to be equally predictive fits the data almost as well as allowing each type of fixation a different weighting—and this indicates that it is just the number of times the top row is fixated compared with the bottom row that matters. Breaking fixations into transitions, it is the between‐row transitions that matter. Transitions ending on the top row increase the probability of choosing the top row, and transitions ending on the bottom row decrease the probability of choosing the top row. Transitions within a row have a weak effect. Importantly, even the rare‐category transitions are associated with choices, even though these do not obviously convey a useful comparison. So, although people do have a tendency to make the common‐category transitions, which suggests that they are using strategically relevant information to make a choice, actually, when using transitions to fit choices, we see that any given uninformative rare‐category transition is just as strongly associated with choice as any given common‐category transition, if not more so. This suggests that while people may be making sensible eye movements, their integration of information is quite simple, with every arrival in a row increasing the probability of choosing that row. This pattern is not consistent with level‐*k* or rational choice models but is consistent with a simple accumulator model of choice where each visit to an alternative is associated with an increased chance of it being chosen.

## Conclusion

For these strategic choices, the choice time and eye movement process data contain the signature effects of accumulator models but are not compatible with level‐*k* or cognitive hierarchy models. First, choices were longer and took more fixations when the payoffs were finely balanced across rows. Second, as a choice unfolds a bias to gaze at the payoffs of the ultimately chosen row emerges. Third, transferring gaze to a row is associated with a higher likelihood of choosing that row. Thus, we argue that processing in strategic decisions, like processing in risky and other multiattribute decisions, is well described as the steady accumulation of evidence over time.

## Instructions

This experiment asks you to make choices while recording your eye movements.

To help the eye tracker, it is useful to use a chin rest to keep your head relatively still.But please do feel free to wriggle between the choices as necessary.

We use a small sticker above your eye to help the eye tracker track and compensate for your head movements.

The next screen will set up the eye tracker. After setting up, you will see the description of task.

[The eye tracker was calibrated.]

Now, the eye tracker is set up.

The next screen explains how to make a choice during the experiment. After these instructions, you will have one practice game. If you are unsure at any time, please ask for clarification.

Press the SPACE to proceed or the ESCAPE to go back.

Each game is a game involving you and involving another participant. Each game has four possible outcomes for you and four possible outcomes for the other participant. What each of you will receive depends on your choice and the other participant's choice. An example is given as follows.

[A screenshot like Figure 1c was shown. The payoffs and choices in the following text were adapted, based on the counterbalancing.]

Your payoff will be one of the numbers in [green/blue] color, and the other player's payoff will be one in [blue/green] color.

Press the SPACE to see the description of this game or the ESCAPE to go back.

Throughout the experiment, you will choose [top/left] or [bottom/right], and the other player selects [left/top] or [right/bottom].

You each make your choice without knowing what the other player has chosen. That is, you do not know what the other person has chosen, and they do not know what you have chosen.

Press the SPACE to proceed or the ESCAPE to go back.

Suppose that you choose [top] in the following example.

Your possible payoffs are [50] and [30], and the other player's possible payoffs are [50] and [60]. Which you obtain will depend upon the other player's selection.

Press the SPACE to proceed or the ESCAPE to go back.

Now, suppose that the other player has chosen [right]. Then, your payoff is [30], and the other player's payoff is [60].

But remember, neither of you can see what the other has chosen. So you will need to think carefully about what you prefer and what you think the other player will do. At the end of the experiment, we are going to pair you up with another participant (chosen at random) and pick out one of the games you have played (again at random). Then we will look up what you each chose. You will each win the outcomes that your joint choices indicate, just like in the example. Each £10 in the experiment is worth £1 for real, so choose carefully. Depending on your choice and the other participant's choice, you can win anywhere from £0 to £9 of real money.

Press the SPACE to proceed or the ESCAPE to go back.

After each game, you will see a “+” in the center of the screen. If you look at it, you will then be taken to the next game.

Press the SPACE to proceed or the ESCAPE to go back.

You will now have a practice choice.

When you decide, please press the [↑/←] or [↓/→] arrow key to indicate your choice.

You can rest your fingers on these keys during the experiment so that you do not need to look at your hand.

Press the SPACE to proceed or the ESCAPE to go back.

[Participants completed a single practice trial.]

This is the end of instructions. If you have any questions or the task is unclear, please ask the experimenter now.

Press the SPACE to start the experiment or the ESCAPE to go back.

## References

[bdm1901-bib-0001] Arieli, A. , Ben‐Ami, Y. , & Rubinstein, A. (2009a). Fairness motivations and procedures of choice between lotteries as revealed through eye movements. Unpublished manuscript.

[bdm1901-bib-0002] Arieli, A. , Ben‐Ami, Y. , & Rubinstein, A. (2009b). Tracking fairness considerations and choice procedures. Unpublished manuscript.

[bdm1901-bib-0003] Arieli, A. , Ben‐Ami, Y. , & Rubinstein, A. (2011). Tracking decision makers under uncertainty. American Economic Journal: Microeconomics, 3, 68–76. doi: 10.1257/mic.3.4.68

[bdm1901-bib-0004] Bogacz, R. , Brown, E. , Moehlis, J. , Holmes, P. , & Cohen, J. D. (2006). The physics of optimal decision making: A formal analysis of models of performance in two‐alternative forced‐choice tasks. Psychological Review, 113, 700–765. 10.1037/0033-295X.113.4.700. 17014301 10.1037/0033-295X.113.4.700

[bdm1901-bib-0005] Busemeyer, J. R. , & Townsend, J. T. (1993). Decision field theory: A dynamic‐cognitive approach to decision making in an uncertain environment. Psychological Review, 100, 432–459. 10.1037/0033-295X.100.3.432. 8356185 10.1037/0033-295x.100.3.432

[bdm1901-bib-0006] Camerer, C. F. , Ho, T. H. , & Chong, J. K. (2004). A cognitive hierarchy model of games. Quarterly Journal of Economics, 119, 861–898. 10.1162/0033553041502225.

[bdm1901-bib-0007] Camerer, C. F. , Johnson, E. J. , Rymon, T. , & Sen, S. (1993). Cognition and framing in sequential bargaining for gains and losses. Frontiers of Game Theory, 104, 27–47. 10.1006/jeth.2001.2850.

[bdm1901-bib-0008] Chen, C.‐T. , Huang, C.‐Y. , & Wang, J. T.‐y. (2011). A window of cognition: Eyetracking the reasoning process in spatial beauty contest games. Unpublished manuscript.

[bdm1901-bib-0009] Costa‐Gomes, M. A. , & Crawford, V. P. (2006). Cognition and behavior in two‐person guessing games: An experimental study. American Economic Review, 96, 1737–1768. 10.1257/aer.96.5.1737.

[bdm1901-bib-0010] Costa‐Gomes, M. A. , Crawford, V. P. , & Broseta, B. (2001). Cognition and behavior in normal‐formal games: An experimental study. Econometrica, 69, 1193–1235. 10.1111/1468-0262.00239.

[bdm1901-bib-0011] Devetag, G. , Di Guida, S. , & Polonio, L. (2015). An eye‐tracking study of feature‐based choice in one‐shot games. Experimental Economics, 10.1007/s10683-015-9432-5.

[bdm1901-bib-0012] Diederich, A. (1997). Dynamic stochastic models for decision making under time constraints. Journal of Mathematical Psychology, 41, 260–274. 10.1006/jmps.1997.1167. 9325121 10.1006/jmps.1997.1167

[bdm1901-bib-0013] Fiedler, S. , & Glöckner, A. (2012). The dynamics of decision making in risky choice: An eye‐tracking analysis. Frontiers in Psychology, 3, 335 10.3389/fpsyg.2012.00335. 23162481 10.3389/fpsyg.2012.00335 PMC3498888

[bdm1901-bib-0014] Funaki, Y. , Jiang, T. , & Potters, J. (2011). Eye tracking social preferences. Unpublished manuscript.

[bdm1901-bib-0015] Horstmann, N. , Ahlgrimm, A. , & Glöckner, A. (2009). How distinct are intuition and deliberation? An eye‐tracking analysis of instruction‐induced decision modes. Judgment and Decision Making, 4, 335–354. Retrieved from http://journal.sjdm.org/9323/jdm9323.pdf

[bdm1901-bib-0016] Hristova, E. , & Grinberg, M. (2005). Information acquisition in the interated prisoner's dilemma game: An eye‐tracking study In Bara B. G., Barsalou L., & Bucciarelli M. (Eds.), Proceedings of the twenty‐seventh annual conference of the Cognitive Science Society (pp. 983–988). Alpha, NJ: Sheridan Printing Retrieved from http://csjarchive.cogsci.rpi.edu/proceedings/2005/docs/p983.pdf

[bdm1901-bib-0017] Johnson, E. J. , Camerer, C. , Sen, S. , & Rymon, T. (2002). Detecting failures of backward induction: Monitoring information search in sequential bargaining. Journal of Economic Theory, 104, 16–47. 10.1006/jeth.2001.2850.

[bdm1901-bib-0018] Knoepfle, D. T. , Wang, J. T. Y. , & Camerer, C. F. (2009). Studying learning in games using eye‐tracking. Journal of the European Economic Association, 7, 388–398.

[bdm1901-bib-0019] Krajbich, I. , Armel, C. , & Rangel, A. (2010). Visual fixations and the computation and comparison of value in simple choice. Nature Neuroscience, 13, 1292–1298. 10.1038/nn.2635. 20835253 10.1038/nn.2635

[bdm1901-bib-0020] Krajbich, I. , & Rangel, A. (2011). Multialternative drift‐diffusion model predicts the relationship between visual fixations and choice in value‐based decisions. Proceedings of the National Academy of Sciences of the United States of America, 108, 13852–13857. 10.1073/pnas.1101328108. 21808009 10.1073/pnas.1101328108 PMC3158210

[bdm1901-bib-0021] Miller, G. A. (1956). The magical number seven, plus or minus two: Some limits on our capacity for information processing. Psychological Review, 63, 81–97. 10.1037//0033-295X.101.2.343. 13310704

[bdm1901-bib-0022] Mosteller, F. , & Nogee, P. (1951). An experimental measurement of utility. Journal of Political Economy, 59, 371–404. 10.1086/257106.

[bdm1901-bib-0023] Mullett, T. L. , & Stewart, N. (2015). Implications of visual attention phenomena for models of preferential choice. Manuscript submitted for publication. 10.1037/dec0000049 PMC5058407 27774490

[bdm1901-bib-0024] Nagel, R. (1995). Unraveling in guessing games: An experimental study. American Economic Review, 85, 1313–1326.

[bdm1901-bib-0025] Noguchi, T. , & Stewart, N. (2014). In the attraction, compromise, and similarity effects, alternatives are repeatedly compared in pairs on single dimensions. Cognition, 132, 44–56. 10.1016/j.cognition.2014.03.006. 24762922 10.1016/j.cognition.2014.03.006

[bdm1901-bib-0026] Petrusic, W. M. , & Jamieson, D. G. (1978). Relation between probability of preferential choice and time to choose changes with practice. Journal of Experimental Psychology: Human Perception and Performance, 4, 471–482. 10.1037/0096-1523.4.3.471.

[bdm1901-bib-0027] Rayner, K. , Pollatsek, A. , Ashby, J. , & Clifton, C. Jr. (2012). The psychology of reading (Second ed.,,). New York: Psychology Press.

[bdm1901-bib-0028] Roe, R. M. , Busemeyer, J. R. , & Townsend, J. T. (2001). Multialternative decision field theory: A dynamic connectionist model of decision making. Psychological Review, 108, 370–392. 10.1037//0033-295X.108.2.370. 11381834 10.1037/0033-295x.108.2.370

[bdm1901-bib-0029] Russo, J. E. , & Dosher, B. A. (1983). Strategies for multiattribute binary choice. Journal of Experimental Psychology: Learning, Memory, and Cognition, 9, 676–696. 10.1037//0278-7393.9.4.676. 10.1037//0278-7393.9.4.676 6227682

[bdm1901-bib-0030] Shimojo, S. , Simion, C. , Shimojo, E. , & Scheier, C. (2003). Gaze bias both reflects and influences preference. Nature Neuroscience, 6, 1317–1322. 10.1038/nn1150. 14608360 10.1038/nn1150

[bdm1901-bib-0031] Stahl, D. O. , & Wilson, P. W. (1994). Experimental‐evidence on players' models of other players. Journal of Economic Behavior & Organization, 25, 309–327. 10.1016/0167-2681(94)90103-1.

[bdm1901-bib-0032] Stahl, D. O. , & Wilson, P. W. (1995). On players models of other players: Theory and experimental‐evidence. Games and Economic Behavior, 10, 218–254. 10.1006/game.1995.1031.

[bdm1901-bib-0033] Stewart, N. (2009). Decision by sampling: The role of the decision environment in risky choice. Quarterly Journal of Experimental Psychology, 62, 1041–1062. 10.1080/17470210902747112. 10.1080/17470210902747112 19306161

[bdm1901-bib-0034] Stewart, N. , Chater, N. , & Brown, G. D. A. (2006). Decision by sampling. Cognitive Psychology, 53, 1–26. 10.1016/j.cogpsych.2005.10.003. 16438947 10.1016/j.cogpsych.2005.10.003

[bdm1901-bib-0035] Stewart, N. , Hermens, F. , & Matthews, W. J. (2015). Eye movements in risky choice. Journal of Behavioral Decision Making. 10.1002/bdm.1854. 10.1002/bdm.1854 PMC4964953 27522985

[bdm1901-bib-0036] Stewart, N. , Reimers, S. , & Harris, A. J. L. (2015). On the origin of utility, weighting, and discounting functions: How they get their shapes and how to change their shapes. Management Science, 61, 687–705. 10.1287/mnsc.2013.1853.

[bdm1901-bib-0037] Stewart, N. , & Simpson, K. (2008). A decision‐by‐sampling account of decision under risk In Chater N. & Oaksford M. (Eds.), The probabilistic mind: Prospects for Bayesian cognitive science (pp. 261–276). Oxford, England: Oxford University Press Retrieved from http://www.stewart.warwick.ac.uk/publications/

[bdm1901-bib-0038] Su, Y. , Rao, L. L. , Sun, H. Y. , Du, X. L. , Li, X. S. , & Li, S. (2013). Is making a risky choice based on a weighting and adding process? An eye‐tracking investigation. Journal of Experimental Psychology: Learning, Memory, and Cognition, 39, 1765–1780. 10.1037/a0032861. 10.1037/a0032861 23687917

[bdm1901-bib-0039] Tanida, S. , & Yamagishi, T. (2010). Testing social preferences through differential attention to own and partner's payoff in a prisoner's dilemma game. Letters on Evolutionary Behavioral Science, 1, 31–34. 10.5178/lebs.2010.8.

[bdm1901-bib-0040] Vlaev, I. , & Chater, N. (2006). Game relativity: How context influences strategic decision making. Journal of Experimental Psychology: Learning, Memory, and Cognition, 32, 131–149. 10.1037/0278-7393.32.1.131. 10.1037/0278-7393.32.1.131 16478346

[bdm1901-bib-0041] Wang, J. T. Y. , Spezio, M. , & Camerer, C. F. (2010). Pinocchio's pupil: Using eyetracking and pupil dilation to understand truth telling and deception in sender‐receiver games. American Economic Review, 100, 984–1007. 10.1257/aer.100.3.984.

[bdm1901-bib-0042] Weibull, J. W. (1995). Evolutionary game theory. Cambridge, MA: MIT Press.

